# Development of an early nurse led intervention to treat children referred to secondary paediatric care with constipation with or without soiling

**DOI:** 10.1186/1471-2431-13-193

**Published:** 2013-11-20

**Authors:** David Tappin, Shazia Nawaz, Caroline McKay, Lorraine MacLaren, Peter Griffiths, Toby A Mohammed

**Affiliations:** 1 Paediatric Epidemiology and Community Health (PEACH) Unit, University of Glasgow, Glasgow, Scotland G3 8SJ, UK; 2 Royal Hospital for Sick Children Glasgow, Glasgow, Scotland G3 8SJ, UK

**Keywords:** Constipation, Child, Intervention studies, Psychological techniques, Medicine

## Abstract

**Background:**

Constipation is a common chronic childhood condition referred to secondary care. Effective treatment requires early intervention, prolonged medication to soften stools and behavioural support to achieve a regular habit of sitting on the toilet to pass a stool. The purpose of this audit and service development was to assess routine consultant paediatrician-led care against minimum standards and if appropriate to develop a nurse-led intervention. The new care package could then be tried out within general paediatric clinics in Glasgow as a service evaluation. NICE guideline (CG99) has a research recommendation to compare nurse-led care with routine consultant-led care.

**Methods:**

Design was an audit then development of a nurse-led intervention followed by a service evaluation. Participants were children (age 0–13 years), referred by their General Practitioner (GP) to the Royal Hospital for Sick Children Glasgow, with constipation the main problem in the GP letter. The audit covered appointment waiting times, intervention provided, initial follow-up and parental satisfaction with routine consultant-led practice. The nurse-led intervention focused on self-help psychology practice with NICE guideline medical support. This was compared with routine consultant paediatrician care in a service evaluation.

**Results:**

The audit found consultant-led care had long waiting times, delayed initial follow-up and variable intervention. The new nurse-led intervention is described in detail. The nurse-led intervention performed well compared with consultant-led care. Less ‘nurse-led’ children, 3/45 (7%), were still constipated passing less than 3 stools per week compared with 8/58 (14%) receiving consultant-led care. Less ‘nurse-led’ parents, 10/45 (22%), reported their child having pain passing stools in the previous week compared with consultant-led care, 26/58 (45%). The proportion of children, over 4 years, free from soiling accidents was similar, 15/23 (65%) in the nurse-led group and 18/29 (62%) with consultant-led care. Parental satisfaction was slightly better in the nurse-led group.

**Conclusion:**

It is difficult to achieve minimum standards using routine consultant-led care for children referred by their GP with constipation. Nurse-led early intervention is feasible and has produced promising results in a service evaluation. An exploratory trial is planned to develop a teaching module, robust outcomes including costs and benefits, and methodology for a definitive trial recommended by NICE.

## Background

Constipation is common in children. Five to thirty percent of the children are constipated at some time depending on the diagnostic criteria [1]. More than one third of children develop chronic symptoms. Constipation is a common reason (4%) for referral of children to secondary care [2]. The exact cause of constipation is not fully understood. Pain is thought to be the most important feature prompting fear and with-holding of passing stools. This leads to chronic symptoms. Eventually incontinence with often overflow of stools, takes place. Incontinence can have devastating psychological sequelae [2]. There is agreement on diagnostic criteria [3]. NICE [2] have established assessment requirements and have systematically reviewed and made recommendations regarding medical treatment.

However, as Sackett described [4], the best external evidence c.f. NICE [2] needs to be integrated with individual clinical expertise in order to realise the best clinical outcomes. This is where the practice of care for children with constipation falls down. Some healthcare professionals underestimate the impact of constipation on the child and family [2]. Children and families are often given conflicting advice. Practice is inconsistent, making treatment potentially less effective and frustrating for all concerned. Children often develop constipation while still in nappies. They are seldom treated or followed up unless there are other features such as blood in the stools from an anal fissure. By the time they reach 3.5-4 years parents are expecting and ‘expected’ to have a child who is ‘toilet trained’. By this time, constipated children have often with-held stools for 2–3 years. They may have already developed treatment resistant or intractable constipation defined as: ‘constipation which does not respond to sustained optimum medical management’ [2]. This may contribute to the often poor clinical outcomes seen in children with constipation [2].

A gap exists in current research evidence which relates to clinical expertise and organization of care. Specifically: What is the required expertise? Who would best provide it? How might that fit into current care pathways? Answering these questions is continuing in the tradition of Spitzer and Sackett in their seminal trial of Nurse Practitioners in Canada in 1971–2 [5]. Of note is that in North America as Sackett states ‘nurse practitioner–initiated care had to include using her associated family physician as an intermediate consultant in caring for her patients’ [5]. Since Spitzer and Sackett’s Burlington Trial, Nurse Practitioners have become important health care professionals in North America, Canada and elsewhere. They work alongside doctors in both primary and secondary care to increase patient capacity while retaining quality and sometimes reducing cost. NICE [2] agree there are gaps in knowledge. NICE have made a specific research recommendation which is: *Do specialist nurse-led children’s continence services or traditional secondary care services provide the most effective treatment for children with idiopathic constipation (with or without faecal incontinence) that does not respond fully to primary treatment regimens? This should consider clinical and cost effectiveness, and both short-term (16 weeks) and long-term (12 months) resolution?* This recommendation focuses on the question: ‘Who would best provide secondary care intervention?’ Our research programme aims also to answer the other two questions: ‘What is the required expertise?’ and ‘How might that fit into current care pathways?’

This report describes the development of a nurse-led *early* intervention within Glasgow for children referred by their General Practitioner with constipation. The project was a feasibility study that included an audit, design of a nurse-led intervention and a service evaluation. The audit of GP referrals to a large secondary care provider over a 3 month period compared care given by consultant general paediatricians to minimum standards [6] of care for children with constipation. A case-note review assessed outcome after 2 years. A nurse-led intervention was designed and piloted, with reference to medical, nursing and psychological practice. Finally there was a service evaluation of the new intervention provided by two ‘nurses’ in Glasgow. This evaluation assessed practicality of the design by working with a number of doctors in one city in general paediatric clinics. It also assessed whether reasonable outcomes resulted, compared with routine care. This report chronologically follows the developments.

This work addresses MRC guidance: Developing and evaluating complex interventions (http://www.mrc.ac.uk/complexinterventionsguidance). It addresses point 3: *Developing a complex intervention*. A clinical trial programme will be needed to evaluate the developed intervention, and to fully address the NICE guideline research recommendation.

## Methods

### Audit

SN a research psychologist performed an audit of all children referred to the Royal Hospital for Sick Children (RHSC) Glasgow with constipation for a three month period from the 1st March to 31st May 2006. A cut-off point for the audit was set as 12th of September 2006. This audit compared the care given with external standards established in 2001 [6]. The pertinent minimum standards were: (a) *appointments* – no child should wait longer than one month between the referral being received and the first appointment being offered, (b) *appointments* – no child should wait longer than 3 months between the referral being received and the actual date of the first appointment, (c) *general follow-up* – follow-up supervisory contact should be within 2 weeks of the first appointment by visit or telephone, (d) *fail to attend* should be sent one further appointment.

The modalities of care given were also described (e.g. history, examination, investigation, medical treatment, behavioural intervention, education).

A modified parent satisfaction questionnaire for children with constipation was also administered [7]. The parent satisfaction scale was adapted from the scale used by Sullivan [7]. The scale covered the following domains: (1) provision of information, (2) empathy, (3) technical quality and competence, (4) attitude towards the patient, (5) access and continuity, (6) overall satisfaction. The satisfaction questionnaire involved parents reading 12 brief statements and responding to them on a 5-point scale. The five points were: strongly agree, agree, not sure, disagree, and strongly disagree. In addition parents were asked 3 open ended questions: What did you like most about your care? What did you like least about your care? Do you have any suggestions for improvements? This questionnaire was sent out to the 17 parents who attended their first appointment. A telephone questionnaire was administered to those who failed to return the written questionnaire.

### Designing a nurse-led intervention, piloting the intervention and teaching it to a nurse

An expert group was formed in 2007. A clinical psychologist (PG) had many years experience treating constipation and soiling. He had expertise in education of parents and child about how the bowel works and what can go wrong [8]. He was also a behavioural therapist [9], instructing parents to help their child to sit on the toilet on a regular daily basis to try to pass a stool. An experienced general paediatrician (DT) had implemented a nurse-led service for nocturnal enuresis [10]. He updated the Cochrane Review of Behavioural and Cognitive Interventions [9] and was a member of the NICE guideline development group for constipation in children – CG99 [2]. He provided expertise on assessment to rule out organic pathology via history and examination and other investigation if required. He also advised on prescription of medication. An experienced children’s nurse and nurse educationalist (TM) helped to develop the nurse-led package of care. SN was a chartered psychologist who had obtained a research PhD supervised by PG and DT, examining new methods of care for night-time wetting [11]. She joined the expert group and undertook the initial audit of GP referrals. SN developed assessment tools and piloted the new intervention within general paediatric clinics run by DT.

This group met on 6 occasions and developed an intervention based on the roles and experience of each group member. Group consensus chaired by DT produced agreement about the final intervention strategy. Experience was included from the pilot phase where SN acted as a ‘nurse’ supervised by DT as the responsible paediatrician.

Funding was provided by the Director of Public Health Glasgow to employ a full time nurse to provide the intervention. CM, an experienced children’s trained nurse and health visitor, was employed and taught the new intervention by SN and DT. This was achieved by direct observation supported by a handbook created by PG, followed by supervision of cases by SN and DT.

Medline, Embase and Cinahl databases 1946/7 to the present, were searched to discover reports of *trial*s of nurse-led services for *child*ren with *constipation,* using the Boolean word AND. The resulting hits were limited to human, English language, constipation in the title, with an abstract available. The titles were read and drug trials, procedural trials such as electrical stimulation, food additive trials, and biofeedback trials were removed. Cinahl produced 9 hits, Embase 9 hits, and Medline 11 hits. The abstracts were read. On reading these abstracts, the only trial comparing nurse-led and doctor-led services was the trial run by Burnett and Sullivan [7,12]. The intervention used by this group was not described in detail in either of these articles but was described in some detail in a supplementary publication [13]. A further literature search was performed as above replacing *trials* with *nurse.* A description of a nurse-led intervention for children with constipation and soiling was called IMPACT [14]. Comparison of IMPACT with the intervention designed by our expert group will be made in the Discussion section.

### Service evaluation

Using SN as a second ‘nurse’ therapist, CM and SN established their own child constipation clinics. These were situated within established outreach general paediatric services. SN and CM were supported by consultant paediatricians who were generally on-site at the same time seeing patients of their own. This model follows the successful nurse led care pathway for night wetting in Glasgow [10]. Glasgow outreach general paediatric services are geographically based usually in large GP run health centres and patients are allocated to them by their postcode of residence. SN and CM were able to cover about half of the outreach general paediatric clinics. This was dependent on the availability of an extra room for the ‘nurse’. SN and CM therefore had regular slots at clinics covering half the city of Glasgow. Children with constipation referred by GPs who lived in the other postcode areas were treated in a routine way by consultant paediatricians alone. These children acted as a comparison group for the new nurse-led service.

All GP general paediatric referrals were secondarily vetted by DT every two weeks over a 7 month period between March and November 2009. Eligible patients were GP referrals, aged 0–13 years, from postcode areas in the City of Glasgow. To be included the main complaint in the referral letter had to be constipation. Other conditions that made a simple nurse-led intervention inappropriate had to be absent e.g. Autistic Spectrum Disorder. Allocation to either the new nurse-led intervention or the comparison group depended on postcode of residence.

Both groups were contacted by SN at least 16 weeks after their first appointment via a structured telephone interview to provide outcome data. The primary outcome was a measure of constipation *less than 3 stools per week*[2,15] for all children, and *soiling in the last week*[15] for children greater than 4 years. Secondary outcomes were: 1. parent satisfaction with the service, 2. still taking medication at follow-up, 3. overall better than prior to first clinic visit, 4. pain passing stools in the last week, 5. with-holding behaviour during the last week, 6. stool that blocked the toilet in the last week. Parent satisfaction was measured in the same way as in the audit, as the average over 12 questions on Likert scales of 1–5 where 1 was always the most positive and 5 the most negative. SN was blind to allocation status prior to follow-up telephone contact unless she had seen the patient herself and the patient remained particularly memorable to her. She remained blind to allocation unless the parent informed her of allocation status during the telephone contact.

Analysis was performed based on a cluster design using both intention to treat and per protocol analysis.

Submission was made to the National Research Ethics Service (NRES) via the *query* facility. Advice indicated that the study was *service evaluation* and as such did not require to be examined by an ethics committee. Further representation to the chairperson of the local ethics committee was concordant with the NRES decision. Consent was not obtained from parents or children as this intervention was being implemented and evaluated as a service development in Glasgow.

## Results

### Audit

Sixty one patients were referred to secondary care in Glasgow with the main problem being constipation over a 3 month period March to May 2006. Case notes of the first 30 patients were reviewed after their initial scheduled appointment. There were 19 boys and 11 girls, mean age 5.1 years range 1.0 to 11.1. Mean area based material deprivation score was 5.0 on a scale from 1 least deprived to 7 most deprived [16]. Fourteen of the subjects had been referred in the past to the general paediatric service with constipation, twelve once, one twice and one three times. Only 21 patients were appointed due to a partial booking system where parents had to phone in to book their appointment once notified by the hospital by letter. Seventeen patients arrived for their first appointment. Seven were treated with medication alone, one was offered behavioural treatment alone (e.g. sitting on the toilet each evening for a small prize), three medication plus behavioural treatment, one education (e.g. how the bowel works and what can go wrong) plus medication and five were given no treatment. Four of the latter five were discharged because the problem had resolved (3) or greatly improved (1). The last given no treatment was referred to the psychology department as the problem was thought to be psychogenic in nature. When the case notes were reviewed in 2008 two years later, 16 of the 30 audited patients had eventually been treated. For 9 there was resolution or significant improvement, 2 were still being treated and for 5 the outcome was unknown as they had defaulted from follow-up.

With regard to the Minimum Standards [6], (a) *Allocation of appointment* was made at a mean of 8.4 weeks with 57% receiving notification of an appointment within the 4 week standard, (b) *M*ean time from referral to appointment was 14.8 weeks with 50% having an appointment within 3 months of referral, (c) *Initial follow-up telephone or clinic* was made at a mean 7.3 weeks only 17% within 2 weeks, (d) All were sent another appointment after *first default*.

Parent satisfaction questionnaires were returned by five of seventeen families and telephone questionnaires were administered to a further four families. Parents were generally satisfied with the service they received with a mean overall score of 1.8 on a range from 1 to 5. Comments about ‘likes’ included ‘helpful’, ‘doctor put me at ease’, ‘nice people’, ‘good advice’, ‘dislikes’ included ‘too quick’, ‘hospital too far away’, ‘no human element’. Suggested improvements included ‘reduced waiting times’, ‘could be friendlier’, ‘clinics in the community’.

### The nurse-led intervention

The expert group developed a nurse-led intervention which relied on close co-operation between the nurse and an experienced doctor [5]. This meant that the ‘nurse’ worked alongside the doctor in a general paediatric clinic.

First appointments were scheduled for one hour, follow-up appointments 30 minutes.

1. History was taken by the nurse using a form developed with the help of a Consultant Paediatric Gastroenterologist. History was reviewed and examination made by a paediatrician to rule out organic pathology.

2. The child and parents were educated about how the bowel works and what can go wrong. Explanation was given that constipation should be treated as a chronic long term condition like asthma which can be managed but seldom cured.

3. The problem was then reframed into a) disimpaction of retained stools followed by effective long term softening of stools with macrogol medication [2] prescribed by the paediatrician and adjusted in liaison with the nurse, and b) small steps to sit on the toilet regularly after an evening meal for 5–10 minutes for a story or a small prize. ‘Blowing bubbles’ was used to help stools pass in younger children, and ‘blowing up balloons’ for older children.

4. To show that ‘self-help [17]’ practice had succeeded, parents and child were formally asked by the nurse at the end of the consultation about their role as trainer (parent) and compliant co-worker (child) and the nurse as remote therapist.

PG developed a nursing manual to act as a textbook and help understanding of psychology practice related to children with constipation.

### Service evaluation

**Figure 1:**
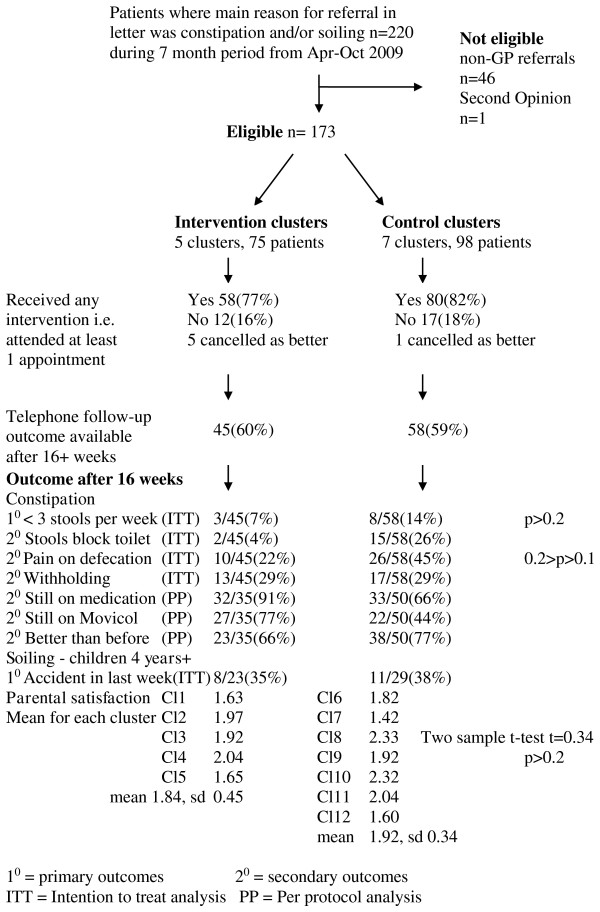
Participant flow diagram.

A participant flow diagram is shown in Figure 1.

Table 1 shows baseline data with no obvious differences between intervention and comparison groups.

**Table 1:** Baseline data

	**Eligible - GP referrals resident in GGHB area**	
	**Nurse-led**	**Consultant-led**
	**75 patients, 5 clusters**	**98 patients, 7 clusters**
Median age at first appointment in years (interquartile range)	3.5 (1.9, 7.3) 0.2-12.4	3.4 (1.9, 7.9) 0.5-15.7
<4 years (%)	40/75 (53%)	56/98 (57%)
Sex M:F	35/40	49/49
Constipation/Constipation + soiling or soiling	52/15 (71%) → n = 67	55/23 (71%) → n = 78
Mean deprivation (range) - 7 most deprived	5.1 (1–7)	4.6 (1–7)
Median length of symptoms in years (interquartile range)	1.9 (0.5, 3.0) → n = 43	1.4 (0.5, 2.7) → n = 66
Median age symptoms began in years (interquartile range)	1.2 (0.6, 3.4) → n = 43	1.2 (0.8, 3.6) → n = 66

Intention to treat analysis included 60% (45/75) of the intervention patients and 59% (58/98) of comparison patients who could be contacted by telephone for outcome assessment after 16 weeks. Per protocol analysis included 47% (35/75) of intervention and 51% (50/98) of the comparison group who had attended for at least one appointment.

The primary outcomes were *passing less than three stools per week,* and *having accidents in the last week,* for children older than 4 years. There was no statistically significant difference between intervention and comparison groups (Table 2), when analysed in keeping with the cluster design. No significant difference was seen for secondary outcomes, which included *passing a stool that would block the toilet*, *having retentive withholding behaviour in last week*, *having painful defecation during last week*, *being better than prior to first clinic visit*, *still taking medication*, and *still taking Movicol*.

**Table 2:** Outcomes comparing nurse-led and consultant-led groups

**Outcomes 16 weeks after first visit**	**Nurse-led**	**Consultant-led**
**Primary outcomes**		
< 3 stools per week	3/45 (7%)	8/48 (14%)
Accident in last week	8/23 (35%)	11/29 (38%)
**Secondary outcomes**		
Stool that blocked toilet in last week	2/45(7%)	15/58 (26%)
With-holding behaviour in last week	13/45 (29%)	17/58 (29%)
Painful defecation during last week	11/45 (24%)	26/58 (45%)
Better than prior to first clinic visit	23/35 (66%)	38/50 (76%)
Still taking laxative medication	32/35 (91%)	33/50 (66%)
Still taking movicol	27/35 (77%)	22/50 (44%)

No significant difference was seen between groups for parental satisfaction (Figure 1).

Lack of engagement with the service was similar between groups. In the intervention group, 5/75 families phoned to cancel as the condition had resolved, 12/70 (17%) did not arrive for any appointments and were discharged, 22/70 (31%) failed to attend their first appointment, and 16/48 (33%) first appointment attendees failed to attend a second appointment. In the comparison group, 1/98 (1%) families phoned to cancel, 17/97 (18%) did not arrive for any appointments and were discharged, 23/97 (24%) failed to attend their first appointment, and 14/74 (19%) first appointment attendees failed to attend a second appointment.

One second opinion was sought in the intervention group.

## Discussion

An audit of routine consultant-led secondary care services for children referred by their GP with constipation, with or without soiling, showed a service model that probably could not reach accepted minimum standards of care [6]. This was particularly related to early follow-up. Intervention provided by doctors was very variable and did not cover all the important areas of history and examination, education, disimpaction and maintenance treatment [14]. A nurse-led model of care was designed by an expert group. Two psychological practices were added to the important areas described above. The first was ‘self-help’ [17], where the parent was trained as the child’s primary therapist and was responsible for administering daily behavioural and medical therapy. The second was ‘reframing’ the problem into two understandable aims: (a) *resolution of pain*, by initial disimpaction of the bowel if required followed by long term softening of the stools and (b) *avoidance of accidents* where possible by developing a regular habit of sitting on the toilet. This new nurse-led early secondary care intervention was subjected to a service evaluation in a large inner city. It was found to be practical and not obviously inferior to routine consultant-led care.

### Audit

The audit of routine doctor-led secondary care indicated areas that needed improvement. This related to speed of notification of appointments and also waiting times which however reflected overall general paediatric waiting times. The follow-up standard of 2 weeks after first appointment was only met in 17% of cases. The accepted requirements [14] of education, disimpaction, maintenance and behavioural therapy, were variably used by paediatricians. Parent satisfaction was encouraging although only collected from just over 50% of parents (9/17). Resolution of waiting times was thought to be possible to meet the minimum standards. The important 2 week follow-up after the first visit was unlikely to happen in a routine consultant-led general paediatric setting. Nurse-led services for elimination disorders had been successful for night-wetting in Glasgow [10]. The audit as well as Royal College of Paediatrician and Child Health guidance [18] supported development of a nurse-led secondary care service for children referred by their GP with constipation. The NICE guideline research recommendations [2] endorsed this development.

### Developing a nurse-led intervention

Some may ask why we didn’t take a nurse-led intervention ‘off the shelf’ that had been piloted elsewhere. There have been nurse-led interventions documented [7,12-14] and used in a small trial [12]. IMPACT [14] is a nurse-led protocol that has many similarities to our intervention. History and examination are described and are similar to the NICE guideline, as is medical treatment which is split into disimpaction and maintenance therapy. Education about how the bowel works and what can go wrong is also clearly described. The main difference is that our intervention explicitly describes the psychological practices of reframing and self-help [17]. CM has attended an IMPACT one day course facilitated by Brenda Cheer. The actual nurse-led process described was akin to our own and emphasized these psychological practices required for effective intervention. This interpretation of the IMPACT intervention was very supportive to our methods and gave us confidence that we were on the right track. The literature review did not provide evidence that IMPACT had been subjected to a randomized controlled trial to examine effectiveness and cost effectiveness. IMPACT was also mainly designed for children with treatment resistant constipation who had failed standard secondary care therapy.

Our hypothesis remains that *early* nurse-led treatment at the point of referral to secondary care will be effective at managing constipation and will reduce the burden of children who fail routine secondary care and develop treatment resistant constipation and the devastating sequelae associated with soiling. This in our view requires a different approach more akin to the methods developed in psychological practice [17] where a parent(s) becomes the child’s therapeutic trainer [19-21]. We also accept that a paediatrician is needed to work alongside the nurse [5] to take responsibility for ruling out organic pathology and to provide credibility for the family. A paediatrician can also support the nurse with initial disimpaction therapy and in situations that may develop such as the need for more aggressive medications, onward referral to Child and Family Psychiatry, Surgery or for more specialist tertiary Gastroenterology care. The intervention developed has been taught to one nurse (CM). She is an experienced children’s nurse who has also been a generic health visitor for many years. She has past experience of using a prescriptive psychological intervention [22]. To teach the intervention to other nurses, a course needs to be developed around the required learning outcomes to provide the intervention [23].

### Service evaluation

What was the service evaluation for? We needed to make sure that our nurse-led intervention was feasible in terms of getting the extra clinic room at general paediatric clinics alongside a paediatrician. It was also important to be sure it was possible to work with a number of different paediatricians and that the nurse-led service could provide acceptable waiting times and early often telephone follow-up. We also wanted reassurance that the new nurse-led early secondary care intervention was not obviously inferior to routine consultant-led secondary care. These aims were achieved and the short-term outcome for constipation after 16 weeks encouraged us to think that a trial of this *early* nurse led intervention may provide evidence of effectiveness and cost effectiveness compared with routine secondary care. ‘Nurse-led’ children were nearly all still taking laxatives at follow-up (91%). This indicates either that the nurse-led therapy was orientated to more prolonged use of laxatives or that treatment compliance was better in the nurse-led group. This difference goes along with improved primary and secondary outcomes.

The next stage will be to run an exploratory trial in two other geographic areas to develop a teaching course for the new intervention and to assess if it allows nurses to provide intervention to a good standard. This exploratory trial will also assess parental and professional views about a new nurse-led secondary care service and develop trial methodology for a definitive trial. The exploratory trial will assess if benefits of the new model of care are likely to outweigh any extra costs.

## Conclusion

We have developed a nurse-led intervention for children who have failed primary care treatment for constipation. This development is in line with the evidence based medicine philosophy established by Sackett [4]. The intervention is practical when used in a deprived inner city and should transfer to other settings. If effective and cost-effective it will change the standard care pathway for children with constipation, from a GP referring to a Paediatrician who may eventually refer treatment resistant cases to a Nurse-led service if one exists, to a GP referring to a Nurse-led service supported by a Paediatrician. The intervention is similar to IMPACT [14] but is designed to utilise psychological practices particularly self-help [17]. Self-help will enable parents to provide early effective intervention supported by a nurse-led team. The aim is to stop children’s constipation becoming treatment resistant. This will reduce both health service costs and the devastating effects of soiling on children and their families. A postgraduate course needs to be developed to teach the intervention [23] which then needs to be subjected to the rigors of a definitive randomised controlled trial. This will fulfil a research recommendation made by the NICE guideline development group [2].

## Abbreviations

RHSC: Royal Hospital for Sick Children, Glasgow.

## Competing interests

The authors declare that they have no competing interests.

## Authors’ contributions

DT conceived the study, negotiated to use funding allocated to child psychology to employ a chartered psychologist (SN) to initially undertake a prospective survey to quantify the size of the problem in Glasgow and outcomes of care both short and long term. He convened an expert group to develop a nurse-led intervention strategy. He then approached the Yorkhill Childrens’ Charity to provide funding to develop and pilot a nurse-led psychological model of care with medical support. PG was a member of the expert group and wrote a manual for nurses to provide a psychological model of care for children with constipation with or without soiling. He reviewed this manuscript. SN undertook the initial survey to document the size of the problem and followed up these patients via case note review. She was part of the service evaluation team and was used as a second ‘nurse’ to provide the nurse-led intervention. She followed up the patients in this service evaluation using a telephone questionnaire. She reviewed this manuscript. CM was the new service nurse, she was taught the intervention by DT and SN and provided the nurse-led intervention and follow-up to more than half of the intervention clusters. She helped vet general paediatric referrals. TM was part of the expert group and supported development of the nurse-led intervention. LM provided administrative support to the expert group, helped vet referrals and provided first line support to parents. All authors read and approved the final manuscript.

## Authors’ information

DT is a consultant general paediatrician and has treated constipation in children for 27 years. He established and runs nurse-led services for other elimination disorders – night [10] and day wetting. He served on the NICE guideline development group for constipation in children (CG99) [2]. He updated the Cochrane review: Behavioural and cognitive interventions with or without other treatments for the management of faecal incontinence in children [9].

SN is a chartered psychologist who gained a PhD by research working on new intervention strategies for children who have night-time wetting [11].

PG is a clinical psychologist who ran clinics for children with elimination disorders in Glasgow and Stirling for many years. He is an author of the Cochrane Review: Behavioural and cognitive interventions with or without other treatments for the management of faecal incontinence in children [9].

TM is a senior nurse responsible for training programmes for nurses.

LM is an administrative assistant who runs secondary care night wetting and constipation services for Glasgow.

## Pre-publication history

The pre-publication history for this paper can be accessed here:

http://www.biomedcentral.com/1471-2431/13/193/prepub

## References

[B1] Benninga MA Voskuijl WP Taminiau JA Childhood constipation: is there New light in the tunnel J Pediatr Gastroenterol Nutr 2004 39 448 464 10.1097/00005176-200411000-00002 15572881

[B2] NICE Constipation in children and young people. Diagnosis and management of idiopathic childhood constipation in primary and secondary care 2010 http://www.nice.org.uk/guidance/CG99 22220325

[B3] Benninga M Candy DCA Catto-Smith AG Clayden G Loening- Baucke V Di Lorenzo C Nurko S The Paris consensus on childhood constipation terminology (PACCT) group J Pediatr Gastroenterol Nutr 2005 40 273 275 10.1097/01.MPG.0000158071.24327.88 15735478

[B4] Sackett DL Rosenberg WMC Muir Gray JA Haynes RB Richardson WS Evidence based medicine: what it is and what it isn’t BMJ 1996 312 71 10.1136/bmj.312.7023.71 8555924 PMC2349778

[B5] Sackett DL A landmark randomized health care trial: the Burlington trial of the nurse practitioner J Clin Epidemiol 2009 62 6 567 570 10.1016/j.jclinepi.2009.01.001 19356898

[B6] Bonner L Dobson P Childhood Soiling: Minimum Standards of Practice for Treatment and Service Delivery: Benchmarking Guidelines 2001 Bristol, UK: ERIC http://www.ericshop.org.uk

[B7] Sullivan PB Burnett CA Juszczak E Parent satisfaction in a nurse led clinic compared with a paediatric gastroenterology clinic for the management of intractable, functional constipation Arch Dis Child 2006 91 499 501 10.1136/adc.2005.087486 16531455 PMC2082804

[B8] Griffiths P The Journey Food Makes http://www.shil.co.uk/Products/the-journey-food-makestm-bowel-training-software.html. Licenced to ERIC (Education and Resources for Improving Childhood continence). Tel 01173012100. Copies available on 27/08/13

[B9] Brazzelli M Griffiths PV Cody JD Tappin D Behavioural and cognitive interventions with or without other treatments for the management of faecal incontinence in children Cochrane Database Syst Rev 2011 12 CD002240 22161370 10.1002/14651858.CD002240.pub4 PMC7103956

[B10] Tappin D Clarke L Ross L Bell M A nocturnal enuresis service for a deprived inner city Acta Paediatr 2003 92 97 102 12650308 10.1111/j.1651-2227.2003.tb00477.x

[B11] Nawaz S Griffiths P Tappin D Parent-administered modified dry-bed training for nocturnal enuresis: evidence for superiority over urine-alarm conditioning when delivery factors are controlled Behav Interv 2002 17 1 14 10.1002/bin.102

[B12] Burnett CA Juszczak E Sullivan PB Nurse management of intractable functional constipation: a randomised controlled trial Arch Dis Child 2004 89 717 722 10.1136/adc.2002.025825 15269068 PMC1720020

[B13] Muir J Burnett C Setting up a nurse-led clinic for intractable childhood constipation BJCN 1999 4 395 399

[B14] Rogers J The IMPACT paediatric bowel care pathway Nurs Times 2008 104 18 46 47 18549102

[B15] van Dijk M Bongers ME de Vries DJ Grootenhuis MA Last BF Benninga MA Behavioral therapy for childhood constipation: a randomized controlled trial Pediatrics 2008 121 e1334 10.1542/peds.2007-2402 18450876

[B16] Carstairs V Morris R Deprivation and Health in Scotland Aberdeen Health Bull (Edinb) 1990 48 4 162 175 2394583

[B17] Hawks D McPherson I, Sutton A The dilemma of clinical practice – surviving as a clinical psychologist in the primary care setting Reconstructing Psychological Practice 1981 London: Croom Helm

[B18] Royal College of Paediatrics and Child Health and the Joint British Advisory Committee on Children’s Nursing Developing Roles of Nurses in Clinical Child Health 1996 London: RCPCH

[B19] Azrin NH Sneed TJ Foxx RM Dry-bed training: rapid elimination of childhood enuresis Behav Res Ther 1974 12 3 147 156 10.1016/0005-7967(74)90111-9 4429525

[B20] McConkey R Jeffree DM Hewson S Involving parents in extending the language development of their young mentally handicapped children Br J Disord Commun 1979 14 3 203 218 10.3109/13682827909011360

[B21] Ryder D Minimal intervention: a little quality for a lot of quantity Behav Change 1988 5 3 100 107

[B22] Tappin DM Lumsden MA McIntyre D McKay C Gilmour WH Webber R Cowan S Crawford F Currie F A pilot study to establish a randomised trial methodology to test the efficacy of a behavioural intervention Health Educ Res 2000 15 4 491 502 10.1093/her/15.4.491 11066466

[B23] Ali A Evans P Multi-resource peer assisted learning (PAL) in postgraduate setting - an enhanced approach. A pilot study J Coll Physicians Surg Pak 2013 23 251 256 23552533

